# Panobinostat PK/PD profile in combination with bortezomib and dexamethasone in patients with relapsed and relapsed/refractory multiple myeloma

**DOI:** 10.1007/s00228-015-1967-z

**Published:** 2015-10-22

**Authors:** Song Mu, Yoshiaki Kuroda, Hirohiko Shibayama, Masayuki Hino, Takeshi Tajima, Claudia Corrado, Rong Lin, Edward Waldron, Florence Binlich, Kenshi Suzuki

**Affiliations:** Novartis Pharmaceuticals Corporation, East Hanover, NJ USA; Hiroshima University Hospital, Hiroshima, Japan; Osaka University Graduate School of Medicine, Osaka, Japan; Osaka City University Hospital, Osaka, Japan; Novartis Pharma KK, Tokyo, Japan; Novartis Pharma AG, Basel, Switzerland; Novartis Pharma S.A.S, Rueil-Malmaison, France; Japanese Red Cross Medical Center, Tokyo, Japan

**Keywords:** Panobinostat, Histone deacetylase inhibitor, Clinical trials, Pharmacokinetics, Pharmacodynamics

## Abstract

**Purpose:**

Panobinostat, a potent pan-deacetylase inhibitor, improved progression-free survival (PFS) in patients with relapsed and refractory multiple myeloma when combined with bortezomib and dexamethasone in a phase 3 trial, PANORAMA-1. This study aims to explore exposure–response relationship for panobinostat in this combination in a phase 1 trial, B2207 and contrast with data from historical single-agent studies.

**Methods:**

Panobinostat plasma concentration–time profiles were obtained in patients from PANORAMA-1 (*n* = 12) and B2207 (*n* = 12) trials. Overall response rates (ORR) and major adverse events (AE) by panobinostat exposure were investigated in the B2207 trial. Panobinostat PK data from combination trials were contrasted with data from single-agent studies.

**Results:**

At maximum tolerated dose (MTD), the geometric mean of panobinostat area under curve from 0 to 24 h (AUC0-24) was 47.5 ng h/mL (77 % CV), and maximum plasma concentration (Cmax) was 8.1 ng/mL (90 % CV). These values were comparable with exposure data obtained in PANORAMA-1, but were 20 % lower than those without dexamethasone, and ∼50 % lower from single-agent trials, likely due to enzyme induction by dexamethasone. Higher levels of panobinostat exposure were associated with higher response rates and higher incidences of diarrhea and thrombocytopenia.

**Conclusions:**

Apparent panobinostat exposure–AE and exposure–ORR relationships were observed when combined with bortezomib and dexamethasone in the treatment of patients with relapsed and refractory multiple myeloma. The addition of dexamethasone facilitated best response even though plasma exposure of panobinostat was reduced. Combination with a strong enzyme inducer should be avoided in future trials to prevent further reduction of panobinostat exposure.

## Introduction

Panobinostat is a pan-histone deacetylase inhibitor (HDACi) with low nanomolar activity against class I, II, and IV histone deacetylases [1, 2]. Panobinostat (Farydak®) in combination with bortezomib and dexamethasone was recently approved in the USA, European Union, and Japan, for treatment of relapsed or relapsed and refractory multiple myeloma, in patients who had received at least 2 prior regimens, including bortezomib and an immunomodulatory agent. The anticancer activity of panobinostat is thought to be due to its effect on epigenetic modulation and inhibition of proteolytic degradation pathways [3, 4]. Panobinostat showed synergistic antimyeloma activity when combined with the proteasome inhibitor, bortezomib, in cell lines in vitro [5, 6], and more recently, this combination showed a clinically relevant extension in progression-free survival in a phase 3 clinical trial (PANORAMA-1), in patients with relapsed and relapsed/refractory multiple myeloma [7]. Previously, an open-label, phase 1b trial (B2207), with escalating doses of panobinostat and bortezomib with or without dexamethasone, was carried out in order to determine the maximum tolerated dose (MTD) and recommended dose for this combination [8]. Both the trials provided data on panobinostat exposure in combination with dexamethasone and bortezomib.

The safety and pharmacokinetic (PK) profile of single-agent panobinostat have been studied in multiple phase 1 and 2 trials [9–12]. Drug metabolism and distribution have been characterized using trace radiolabeled panobinostat [10]. Other studies addressed changes in panobinostat PK or safety in patients with impaired hepatic or renal function [13, 14], interactions of panobinostat with a sensitive CYP2D6 substrate, a strong CYP3A inhibitor, and effect of food on panobinostat disposition [15–17].

The objective of the current study is to characterize the PK of panobinostat in the combination regimen with dexamethasone and bortezomib (PANORAMA-1 and B2207 clinical trials [7, 8]), in comparison with the PK profile of the single agent. The relationship of panobinostat exposures to overall response rate (ORR) per International Myeloma Working Group (IMWG) criteria and frequency of occurrence of grade 3 or 4 adverse events (AEs) in the phase 1b trial B2207 were also analyzed.

### Clinical pharmacology of single-agent panobinostat

**Fig. 1:**
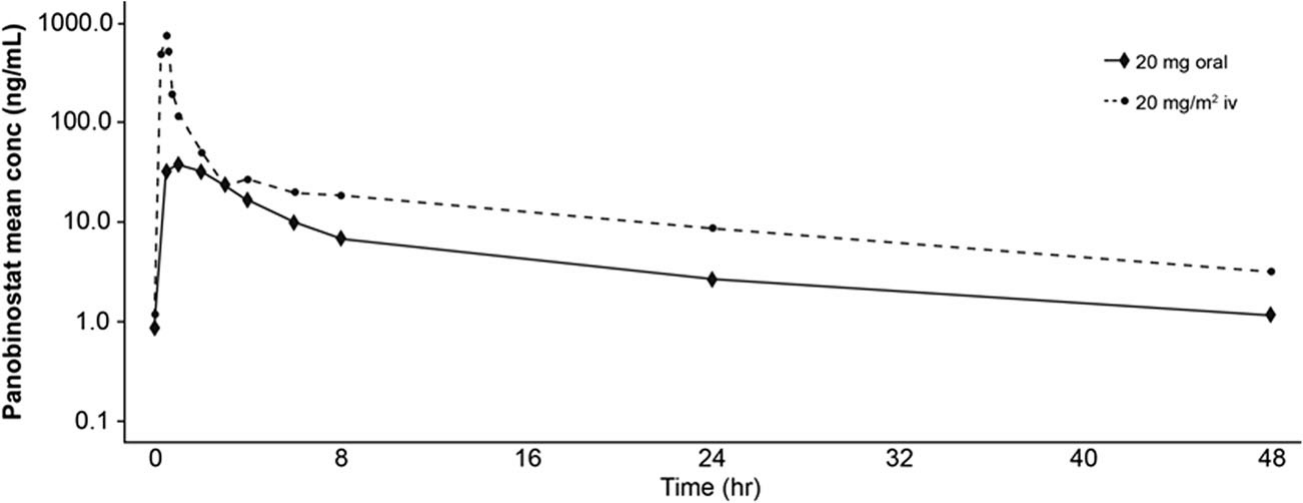
Plasma concentration-time profiles of panobinostat following a single 20 mg/m^2^ IV dose and a single 20 mg oral dose

Panobinostat is rapidly absorbed following oral administration with a median time (Tmax) to reach maximum plasma concentration (Cmax) of about 2 h. Mean concentration–time profiles after a 20 mg/m^2^ iv or 20 mg oral administration can be found in Fig. 1 [10]. The effective half-life (T1/2) of panobinostat is approximately 16 h based on the rate of accumulation of approximately 1.1-fold observed in single-agent trial [10], and steady state is reached after the third dose in a thrice-weekly (tiw) dosing schedule [10]. It is extensively metabolized through both CYP and non-CYP mediated pathways; the fraction of the radiolabeled dose recovered in the feces and urine was 44 to 77 % and 29 to 51 %, respectively [10]. Unchanged panobinostat in the feces accounted for <3.5 % of the administered dose, suggesting good oral absorption [10]. Pharmacokinetics of panobinostat is approximately linear in the tested dose range between 10 and 30 mg. The oral bioavailability of panobinostat is marginally affected by fed or fasting state of the patient, and the food effect is not considered clinically relevant [17].

Panobinostat is biotransformed into over 70 metabolites, none of which showed pharmacologic activity at concentrations of up to 30 μM [10]. Panobinostat shows a weak drug–drug interaction potential. Ketoconazole, a strong CYP3A inhibitor, increased the systemic exposure of panobinostat by 80 % in cancer patients [16]. Panobinostat increased the systemic exposure of a sensitive CYP2D6 substrate, dextromethorphan by 60 %, in cancer patients [15]. Mild, moderate, or severe renal impairment did not alter the plasma exposure of panobinostat in patients with solid tumors [14]; however, mild and moderate hepatic impairment increased panobinostat exposure by 43 and 105 %, respectively [13].

## Methods

Multiple clinical trials with panobinostat PK are compared in the current analysis. All patients in the trials gave written informed consent for participation, and the study protocols were approved by the institutional review board/independent ethics committee/research ethics board at each study site. The clinical trials PANORAMA-1 [7] and B2207 [8] are registered at *ClinicalTrials.gov* with the identifier numbers NCT01023308 and NCT00532389, respectively. A detailed description of the study design and key results for panobinostat single-agent trials are available in the published literature [9, 11, 12, 18].

### Study design

#### B2207 clinical trial

This was an open-label, phase 1b study done in 2 phases: a dose-escalation phase (using 10–30 mg oral panobinostat and 1 or 1.3 mg/m^2^ intravenous [IV] bortezomib) to determine MTD and a dose-expansion phase at MTD to determine safety and efficacy. The MTD was 20 mg tiw panobinostat and 1.3 mg/m^2^ twice-weekly (biw) bortezomib. In the dose-expansion phase, patients were treated at MTD for eight, 3-week cycles (2 weeks of panobinostat and bortezomib therapy and 1 week with no drugs). In addition, 20-mg oral dexamethasone was added starting cycle 2 on the day of and the day after bortezomib therapy. After 8 cycles, patients could discontinue bortezomib/dexamethasone and continue on panobinostat alone until disease progression or discontinuation.

#### PANORAMA-1 clinical trial

This was a phase 3, randomized, double-blind, placebo-controlled trial that assessed efficacy of panobinostat versus placebo plus bortezomib and dexamethasone at the MTD dose and schedule, determined in B2207 trial, in 2 treatment phases. Each phase was of 24 weeks duration with treatment phase 1 consisting of eight, 3-week cycles and treatment phase 2 consisting of four, 6-week cycles. Those patients who had received clinical benefit in treatment phase 1 could proceed to treatment phase 2, in which the bortezomib dose schedule was dropped to once weekly (qw) instead of biw.

### PK assessment

#### B2207 trial

PK assessment was carried out in the dose escalation and dose expansion parts of the trial for both panobinostat and bortezomib. In the dose escalation part, PK samples were collected on cycle 1, day 8, and cycle 1, day 15 at pre-dose, and various intervals post-dose up to 48 h. In the dose expansion part, an initial PK sampling was done on cycle 1 day 8 to obtain PK data for panobinostat and bortezomib in the absence of dexamethasone. Subsequent PK sampling was done on cycle 2, day 8 to obtain PK data for panobinostat and bortezomib in the presence of dexamethasone. Concentration–time profiles of dexamethasone were not assessed. Blood samples were taken at pre-dose (for both drugs): at 5 and 15 min (for bortezomib alone) and at 0.5, 1, 2, 3, 4, 6, 8, 24, and 48 h post-dose (for both drugs). Plasma panobinostat concentrations were measured using a validated liquid chromatography tandem mass spectrometry (LC-MS/MS) method with lower limit of quantification (LLOQ) of 0.1 ng/mL [10, 19].

#### PANORAMA-1 trial

In this study, plasma samples for panobinostat and bortezomib were collected in a subset of Asian patients. Blood samples were taken at scheduled time points (cycle 1, day 1, and cycle 1 day 8 at pre-dose and at 0.5, 1, 2, 3, 4, 8, 24, and 48 h post-dose) for PK analysis.

### Pharmacokinetic analysis

Pharmacokinetic analyses were conducted in WinNonlin Pro version 5.2 (Pharsight, Gary, NC) to derive PK parameters such as Cmax and Tmax for panobinostat and bortezomib, total body clearance (CL/F), apparent volume of distribution (Vz/F), area under the curve from time zero to 24 h (AUC0-24), area under the curve from time zero to infinity (AUC0-inf), and terminal half-life (T1/2).

### Efficacy and safety assessment

The two combination studies, B2207 and PANORAMA-1, reported efficacy data in patients with relapsed and refractory multiple myeloma [7, 8]. In the current analysis, exposure–efficacy relationship was explored using the efficacy data (ORR based on IMWG criteria) from the B2207 trial. Exposure–safety relationship was also characterized for the dose ranges of 10 to 30 mg panobinostat and 1 or 1.3 mg/m^2^ bortezomib in the B2207 trial.

## Results

The baseline demographic data for the subset of patients with PK information from PANORAMA-1 and B2207 clinical trials are summarized in Table 1. The median age for patients in the PANORAMA-1 trial was 63 years and for patients in the B2207 was 62 years. The B2207 trial had 19 centers in Australia, Europe, and North America [8], and the PANORAMA-1 study had 215 centers in 34 countries, including Japan [7]. The PK data for PANORAMA-1 study were provided only from centers in Japan. Both trials enrolled patients with a median of 2 prior therapies, and ≥50 % of patients had bortezomib in their prior line of therapy.

**Table 1:** Patient characteristics and baseline demographics for B2207 and PANORAMA-1 studies

Demographic or clinical characteristics	B2207 (dose-expansion phase)	PANORAMA-1 (Japan subset)
	PAN + BTZ + Dex	PAN + BTZ + Dex
	*N* = 15	*N* = 18
Median age in years (range)	62 (48–71)	63.5 (41–75)
ECOG PS		
0 *n* (%)	12 (80 %)	13 (72.2 %)
1 *n* (%)	3 (20 %)	5 (27.8 %)
Prior therapies median (range)	2 (1–7)	2 (1–3)
Prior therapy		
Prior BTZ, *n* (%)	11 (73.3 %)	9 (50 %)
Refractory to BTZ, *n* (%)	4 (26.7 %)	NA
Prior lenalidomide, *n* (%)	7 (46.7 %)	2 (11.1 %)
Prior thalidomide, *n* (%)	5 (33.3 %)	3 (16.7 %)
Prior melphalan, *n* (%)	1 (6.7 %)	16 (88.9 %)
Prior ASCT, *n* (%)	14 (93.3 %)	NA

*ASCT* autologous stem cell transplant, *PAN* panobinostat*, BTZ* bortezomib, *Dex* dexamethasone, *ECOG PS* Eastern Cooperative Oncology Group Performance Status score

### Dexamethasone effect on panobinostat exposures

B2207 study used escalating doses of panobinostat (10, 20, 25, and 30 mg) in combination with 1.0 or 1.3 mg/m^2^ bortezomib. In the dose-expansion phase, 20 mg panobinostat plus 1.3 mg/m^2^ bortezomib was used in the absence (cycle 1, day 8) or presence (cycle 2, day 8) of 20 mg dexamethasone. Pharmacokinetic parameters from B2207 trial are summarized in Table 2. Geometric mean (percent coefficient of variation, CV) of panobinostat exposure (AUC0-24) determined in the absence of dexamethasone on cycle 1 day 8 was 61.8 (60.9 %) ng h/mL and in the presence of dexamethasone (cycle 2, day 8), in the same patient population was 47.5 (76.8 %) ng h/mL. The maximum plasma concentration (Cmax) of panobinostat on cycle 1 day 8 in the absence of dexamethasone was 9.5 (60.4 %) ng/mL and in the presence of dexamethasone on cycle 2, day 8 was 8.1 (90.3 %) ng/mL. In the presence of dexamethasone, an approximately 20 % lower panobinostat exposure was observed, with no apparent difference in the half-life (Table 3).

**Table 2:** PK parameters for panobinostat in B2207 trial

PK parameters	Cycle 1 day 8 (*n* = 15) in the absence of Dex	Cycle 2 day 8 (*n* = 12) in the presence of Dex
AUC0-24 (ng h/mL)	61.8 (60.9)	47.5 (76.8)
Cmax (ng/mL)	9.5 (60.4)	8.1 (90.3)

**Table 3:** Panobinostat PK parameters for single-agent studies vs combination studies

PK parameters	20 mg PAN single agent (tiw qw) *N* = 32	B2207 cycle 2 day 8 PAN + BTZ + Dex *N* = 12	PANORAMA-1 cycle 1 day 8 PAN + BTZ + Dex *N* = 12
Tmax (h)	1 (0.5–8)	1 (0.5–6.3)	2.02 (0.5–4.0)
Cmax (ng/mL)	21.6 (83)	8.1 (90.3)	15.3 (39.0)
AUC0-24 (ng h/mL)	139 (71)	47.5 (76.8)	95.2 (28.4)
T1/2 (h)	16.9 (33)	15.9 (29.2)	16.7 (21.0)
CL/F (L/h)	99.8 (53)	285.2 (79.4)	147.6 (30.8)
Vz/F (L)	2337 (53)	6539 (81)	3553 (32)

Values are median (range) for Tmax and geometric mean (% coefficient of variation, CV) for all other parameters

*N* is the number of patients having an eligible record of multiple oral dose, *AUC* area under the curve, *CL/F* oral clearance, *Cmax* maximum plasma concentration, *Tmax* time to reach Cmax, *T1/2* half-life of panobinostat, *Vz/F* apparent volume of distribution

**Fig. 2:**
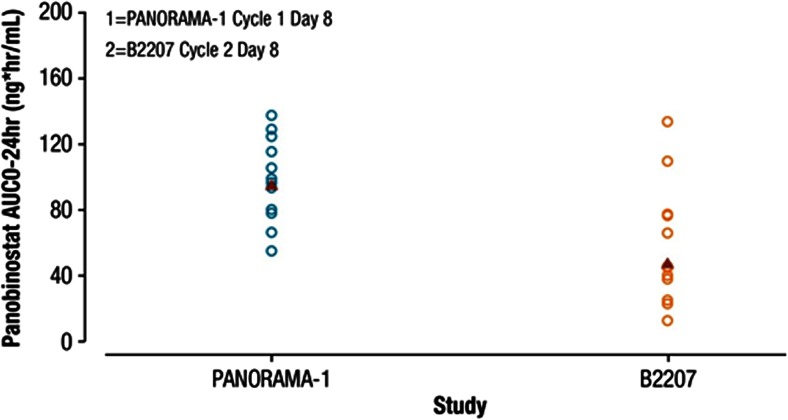
Exposure comparison between PANORAMA-1 and B2207 patients

In single-agent studies [12, 18], PK data were taken from patients having an eligible record of multiple oral doses of 20 mg (tiw) panobinostat for comparison. Table 3 shows that the T1/2 (16 h) and Tmax (1–2 h) of panobinostat are the same between historical single-agent trials and the 2 combination trials. However, lower panobinostat exposures (AUC0-24) were seen in both B2207 and PANORAMA-1 compared to single-agent studies. In the B2207 study, the geometric mean of AUC0-24 was 48 (77 %) ng·h/mL, and in PANORAMA-1 study, it was 95 (28 %) ng·h/mL, with the range of values largely overlap (Fig. 2). In single-agent studies, the geometric mean (% CV) of AUC0-24 was higher at 139 (71 %) ng h/mL.

### Relationship between panobinostat exposures and response in the B2207 study

**Fig. 3:**
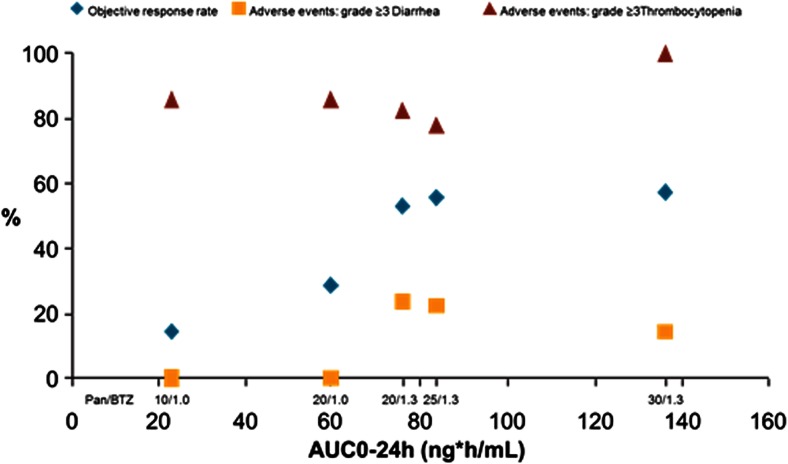
Exposure-response relationship – B2207

A summary of efficacy data and PK parameters (AUC0-24 and Cmax) for individual doses of panobinostat is given in Table 4. The rates of ORR and grade 3 or 4 diarrhea and thrombocytopenia are plotted against panobinostat exposures for individual doses of panobinostat in Fig. 3. In the B2207 study, during dose-escalation phase, no dexamethasone was administered to patients. The ORR defined as greater than or equal to partial response (PR) increased with increasing dose of bortezomib (from 1.0 to 1.3 mg/m^2^) and panobinostat (10–30 mg). Two (28.6 %) patients showed a response of ORR ≥ PR at the dose of 20 mg panobinostat and 1 mg/m^2^ bortezomib, whereas 9 (52.9 %) patients achieved ORR ≥ PR upon increasing the dose of bortezomib from 1 to 1.3 mg/m^2^ and keeping panobinostat dose constant at 20 mg. The number of patients with ORR ≥ PR was 11 (73.3 %) for the group that received 20 mg dexamethasone in addition to the combination of panobinostat 20 mg and bortezomib 1.3 mg/m^2^ (Table 4). ORR appears to plateau at the dose of 20 mg (tiw) panobinostat and 1.3 mg/m^2^ (biw) bortezomib in the dose-escalation phase (Fig. 3).

### Association of panobinostat exposures with thrombocytopenia in the B2207 study

During the dose-escalation phase of the B2207 study, incidences of grade 3 or 4 thrombocytopenia were >80 % (Table 4 and Fig. 3). During the dose-expansion phase (20 mg tiw panobinostat and 1.3 mg/m^2^ biw bortezomib) that used a 2-week dosing schedule and 1-week rest with no drugs, the incidence of grade 3 or 4 thrombocytopenia dropped to 66.7 %. In the dose-escalation phase, the rate of grade 3 or 4 thrombocytopenia (85.7 %) was the same for 10 mg (tiw) and 20 mg (tiw) panobinostat dose at a constant bortezomib dose of 1.0 mg/m^2^ (biw). At the higher bortezomib dose of 1.3 mg/m^2^ (biw), the rates of grade 3 or 4 thrombocytopenia were 82.4 % (20 mg tiw panobinostat), 77.8 % (25 mg tiw panobinostat), and 100 % (30 mg tiw panobinostat), respectively (Table 4 and Fig. 3).

### Association of panobinostat exposures with diarrhea in the B2207 study

In the B2207 study dose-escalation phase, at the lower bortezomib dose of 1.0 mg/m^2^ biw and 10 mg or 20 mg tiw panobinostat, no grade 3 or 4 diarrhea was seen (Table 4 and Fig. 3). At the higher bortezomib dose of 1.3 mg/m^2^ biw, the incidences of grade 3 or 4 diarrhea were 23.5, 22.2, and 14.3 % for the panobinostat doses of 20, 25, and 30 mg tiw, respectively. The absence of grade 3 or 4 diarrhea with 20 mg panobinostat and 1.0 mg/m^2^ bortezomib (*n* = 7) and their emergence (23.5 %) with 20 mg panobinostat and 1.3 mg/m^2^ bortezomib (*n* = 17) implicates bortezomib’s contribution in this particular AE, even though the observation was based on a very small sample size. During the dose-expansion phase that used 2 weeks on and 1 week off dosing schedule with 1.3 mg/m^2^ bortezomib, the rate of grade 3 or 4 diarrhea was 20 %.

Key grade 3 or 4 AEs from B2207, PANORAMA-1 combination studies and single-agent studies are summarized in Table 5. Though panobinostat exposure is lower in the 2 combination studies, the toxicities are higher. For example, grade 3 or 4 thrombocytopenia occurrence in single-agent studies was about 21 %, whereas it was 81 % in the B2207 study and 57 % in the PANORAMA-1 study. Grade 3 or 4 diarrhea was about 3 % in single-agent studies, whereas it was 16 % in the B2207 study and 26 % in the PANORAMA-1 study. The difference in the rates of AEs in the combination studies compared to single-agent studies could be attributed to overlapping toxicities with bortezomib (Table 5).

**Table 4:** Summary of data on efficacy, exposure, and grade 3 or 4 adverse events (thrombocytopenia and diarrhea) in the B2207 study

Efficacy	Dose-escalation phase without Dex; PAN given thrice weekly					Dose expansion
	PAN 10 mg + BTZ	PAN 20 mg + BTZ 1.0	PAN 20 mg + BTZ 1.3	PAN 30 mg + BTZ 1.3	PAN 25 mg + BTZ 1.3	PAN 20 mg (2 weeks on/1 week off) + BTZ 1.3 mg/m^2^ + D × 20 mg
	1.0 mg/m^2^	mg/m^2^	mg/m^2^	mg/m^2^	mg/m^2^	
	*N* = 7	*N* = 7	*N* = 17	*N* = 7	*N* = 9	*N* = 15
AUC0-24 (ng h/mL) cycle 1 day 8	24.1 (33.0)	87.5 (100.2)	89.1 (58.8)	102.8 (45.0)	106.4 (31.6)	61.8 (60.9)
Cmax (ng/mL) cycle 1 day 8	3.5 (10.8)	10.8 (125.5)	15.8 (63.2)	14.5 (74.8)	18.0 (47.6)	9.5 (60.4)
Grade 3/4 thrombocytopenia, *n* (%)	6 (85.7)	6 (85.7)	14 (82.4)	7 (100)	7 (77.8)	10 (66.7)
ORR n (%) and 95 % CI	1 (14.3)	2 (28.6)	9 (52.9)	4 (57.1)	5 (55.6)	11 (73.3)
	[0.4, 57.9]	[3.7, 71]	[27.8, 77.0]	[18.4, 90.1]	[21.2, 86.3]	[44.9, 92.2]
Grade 3/4, diarrhea, *n* (%)	0	0	4 (23.5)	1 (14.3)	2 (22.2)	3 (20.0)

Values are given as geometric mean (% CV) for AUC0-24 and Cmax

*ORR* objective response rate

**Table 5:** Selected grade 3 or 4 adverse events from single-agent studies (pooled patient population) vs B2207 and PANORAMA-1 combination trials

Preferred term Adverse events	20 mg PAN (tiw qw) (*N* = 309) n (%)	B2207 All Patients (10–30 mg PAN) + 1.0 or 1.3 mg/m^2^ BTZ dose (*N* = 62) *n* (%)	PANORAMA-1 20 mg PAN + 1.3 mg/m_2_ BTZ+ 20 mg Dex (*N* = 381) *n* (%)
Anemia	29 (9)	11 (18)	63 (17)
Neutropenia	42 (14)	17 (27)	92 (24)
Thrombocytopenia	66 (21)	50 (81)	217 (57)
Diarrhea	10 (3)	10 (16)	97 (26)
Nausea	6 (2)	0	21 (6)
Vomiting	4 (1)	3 (5)	28 (7)
Fatigue	13 (4)	7 (11)	65 (17)

## Discussion

Three different analyses of panobinostat exposure-response derived from 2 clinical trials were carried out. Initially, the effect of 20 mg dexamethasone on panobinostat exposure was analyzed. Next, the association of panobinostat exposure to rate of response was assessed, and finally, the association of panobinostat exposure to rates of key AEs was determined.

### Dexamethasone effect

**Fig. 4:**
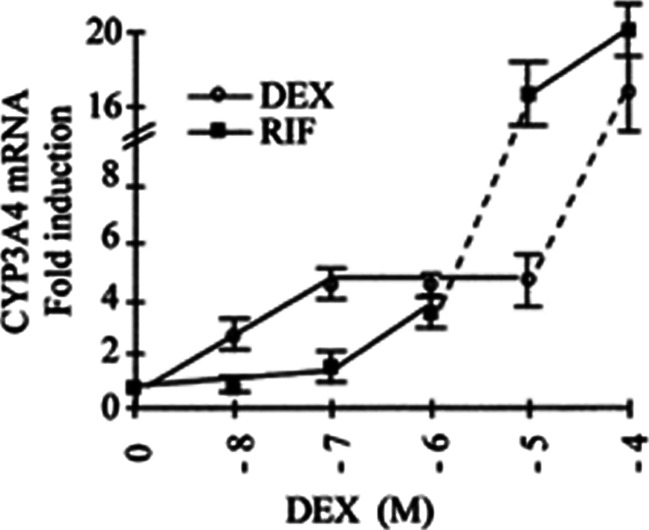
Biphasic induction of CYP3A4 by dexamethasone. *CYP3A4* cytochrome P450, isoform 3A4, *DEX* dexamethasone, *RIF* rifampicin a linear inducer of CYP3A4. Fig. 4 is adapted with permission from Pascussi, JM, et al. (2001) *Eur J Biochem* 268:6346–6358

A prior study in healthy volunteers showed that 8 mg oral dexamethasone given twice daily for 5 days caused an increase in CYP3A4 activity by approximately 25 % [20]. Isolated hepatocyte cultures treated with increasing concentrations of dexamethasone (2–250 μM) showed an increase of CYP3A4 activity from 1.7 to 6.9-fold [20]. Another study in primary human hepatocytes in culture showed a biphasic induction of CYP3A4 mRNA due to dexamethasone [21]. At low concentrations (below 100 nM), dexamethasone induces the nuclear protein pregnane X receptor expression, and this in turn transactivates CYP3A4 mRNA. At higher concentrations (up to 100 μM), dexamethasone directly activates the pregnane X receptor, with a concurrent increase in CYP3A4 mRNA [21]. This is represented in Fig. 4 (adapted with permission). At the 20 mg oral dose of dexamethasone used in the B2207 study, the projected Cmax for dexamethasone is approximately 30 nM. Another pharmacokinetic study using 20 mg panobinostat in combination with 25 mg lenalidomide and 40 mg dexamethasone (day 1 through day 4) showed a further decrease of approximately 60 % in PAN exposure compared to historical single-agent data (unpublished data). Since the fraction of panobinostat metabolized through CYP3A is approximately 40 % [10], it is susceptible to inducers of CYP enzymes including dexamethasone. The reduction of panobinostat plasma exposure in combination with dexamethasone confirmed this hypothesis [10].

In the current study, both the combination trials (B2207 and PANORAMA-1) reported lower levels of panobinostat plasma exposure than historical single-agent studies through reduced bioavailability that should be the result of increased first-pass metabolism. Thus, drug–drug interaction with dexamethasone needs to be considered for drugs whose major metabolic pathways involve cytochrome P450 enzymes in clinical trials to avoid exposing patients to subtherapeutic doses since dexamethasone is the backbone therapy in many combinations for multiple myeloma.

### Exposure-response analysis

In the dose escalation part of the B2207 trial, the ORR increased with the increase in bortezomib or panobinostat exposure. Maximum ORR for panobinostat/bortezomib combination (77.3 %) was achieved upon addition of dexamethasone in the dose-expansion phase. This was in spite of a change in dosing schedule (2 weeks on and 1 week off).

It is worth noting that ORR-AUC relationship in the escalation phase of B2207 trial appears to follow a sigmoidal Emax curve. However, there was an approximately 20 % increase in ORR when 20 mg panobinostat was combined with 1.3 mg/m^2^ bortezomib, instead of 1.0 mg/m^2^ (Fig. 3). A lower dose of panobinostat (10 mg) with 1.3 mg/m^2^ of bortezomib is planned to be tested in future trials to see if it could increase the response as the data suggests. The dose of 10 mg panobinostat administered tiw in combination with 1.3 mg/m^2^ bortezomib may provide a unique balance between efficacy and toxicity.

### Analysis of exposure and key toxicities

Thrombocytopenia is a known toxicity of most pan-histone deacetylase inhibitors and may compromise the options for combination therapies in cancer patients. In the 2 combination trials, the dose of bortezomib impacts the rate of severe thrombocytopenia. In the PANORAMA-1 trial, as subset analysis of 102 patients who completed both treatment phases of panobinostat plus bortezomib plus dexamethasone therapy, the occurrence of grade ≥3 thrombocytopenia reduced from 47 % in treatment phase 1 (that used twice weekly bortezomib dose), to 6 % in treatment phase 2 (that used once weekly bortezomib dose) [22]. An earlier dose-escalation study of panobinostat alone in patients with advanced hematologic malignancies reported 55 % grade ≥3 thrombocytopenia [12]. Recent data suggest that thrombocytopenia may be a down-stream event of histone acetylase inhibition at bone marrow [23]. Thus, thrombocytopenia may be used as a biomarker for target inhibition and dose/regimen optimization.

In patients treated with bortezomib alone, data indicate that incidences of diarrhea were sensitive to bortezomib Cmax. An analysis by Moreau et al. of IV vs subcutaneous (SC) treatment of single-agent bortezomib in patients with multiple myeloma showed that diarrhea is sensitive to bortezomib Cmax [24]. Patients treated with 1.3 mg/m^2^ SC bortezomib had a lower mean (SD) Cmax 20.4 (8.87) ng/mL, and 2 % grade ≥3 diarrhea, whereas patients treated with 1.3 mg/m^2^ IV bortezomib had higher mean (SD) Cmax 223 (101) ng/mL, and 5 % grade ≥3 diarrhea [24]. Diarrhea is one of the overlapping toxicities between bortezomib and panobinostat and is sensitive to bortezomib exposure. Decrease in incidences of diarrhea was seen in the PANORAMA-1 trial upon reducing bortezomib dose to once weekly schedule [25]. Thus, future clinical management of key AEs may be facilitated by dose adjustments and dose reductions.

This study summarizes key analyses of panobinostat pharmacokinetics that will be useful in the treatment of patients with relapsed and refractory multiple myeloma. To date, pharmacokinetics of panobinostat in combination with bortezomib and dexamethasone is very limited, and exposure–response relationship of panobinostat in this combination has not been published. Given the constrains of limited exposure, this study attempted to demonstrate that dexamethasone, which is the backbone of most therapeutic regimens for multiple myeloma, reduces the plasma concentration of panobinostat, 40 % of which is metabolized by CYP 3A4 enzyme. An alternative regimen of once weekly bortezomib is being increasingly used in clinical practice and, at present, represents the standard schedule recommended for elderly patients leading to corresponding change in dexamethasone dose and schedule. Characterization of panobinostat PK in such combinations will be necessary to enable the establishment of exposure–response correlation and provide clinical guideline on its use. This study highlights the importance of understanding drug–drug interactions in combination therapy regimens. Early considerations to exposure response relationships would aid the design of pivotal phase 3 trials.
